# Supplementary material for: Magnitude and determinants of adequate antenatal care service utilization among mothers in Southern Ethiopia
Source: PLoS One. 2021 Jul 6;16(7):e0251477. doi: 10.1371/journal.pone.0251477 (PMC8259961; doi:10.1371/journal.pone.0251477)
Supplement: S1 File — (DOCX) [file pone.0251477.s001.docx]

**Structured questionnaire used for data collection**

**Name of sub city_________________Name of Kebele_____________Household ID______**

| **Section 1: Socio-demographic and reproductive characteristics of participants** | | | |
| --- | --- | --- | --- |
| - 1. **Socio-demographic characteristics of participants** | | | |
| 101 | How old are you? | age in completed years |  |
| 102 | What is the highest level of formal education you have completed? | 1. No formal education 2. Primary education (1-8) 3. Secondary education (9-12) 4. Teritiary and above (12^+^) |  |
| 103 | What is your main occupation? | 1. Not employed (Have no job) 2. Government employee 3. Non-governmental employee 4. private employee 5. self-employee 6. Other (specify) |  |
| 104 | What is your religion? | 1. Orthodox 2. Protestant 3. Adventist 4. Catholic 5. Muslim 6. Other |  |
| 105 | What is your marital status? | 1. currently married 2. cohabiting/living together 3. Never married 4. Widowed 5. Divorced 6. Separated | Skip to Q 108 |
| 106 | What is your husband’s occupational status? | \| 1. Not employed \| \| \| \| \| \| --- \| --- \| --- \| --- \| --- \| \| 1. Government employee \| \| \| \| \| \| \| \| 1. Non-governmental Organization \| \| \| \| \| \| \| \| \| 1. Private employee \| \| \| \| \| \| \| 1. Self-employed \| \| \| \| \| \| 1. Other (specify) \|  \|  \|  \| |  |
| 107 | What is the highest level of formal education your husband completed? | 1. No formal education 2. Primary education (91-8) 3. Secondary education (9-12) 4. Tertiary and above (12^+^) |  |
| 108 | Is there any health professional in your close family/household? | 1. yes 2. No |  |
| 109 | How often did you watch/listen or use (TV/Radio/internet)? | 1. Almost every day 2. At least once a week 3. Less than once a week 4. Not at all |  |
| 110 | Who made the final decisions on maternal health care demand in your household? | \| 1. Respondent alone \| \| --- \| \| 1. Husband /partner alone 2. Respondent and partner jointly \| \| \| 1. Parents 2. Other (specify) \| \| |  |
| 111 | Who is the head of the household? | \| 1. Respondent alone \| \| --- \| \| 1. Husband/ partner alone \| \| \| 1. parents 2. Other (specify) \| \| |  |

| 1**.2. Reproductive characteristics of participants** | | | | |
| --- | --- | --- | --- | --- |
| 112 | | How old were you when you became pregnant with your last birth? | ______________ |  |
| 113 | | How many total numbers of pregnancies do you have in your life time? | ________________ |  |
| 114. | | How many total numbers of deliveries do you have in your life time? | _______________ |  |
| 115. | | How many children do you have currently? | _______________ |  |
| 116 | | How many total numbers of abortions do you have in your life time? | ________________ |  |
| 117 | | How many total numbers of stillbirths do you have in your life time? | ________________ |  |
| 118 | | What is the birth order of the last child? | 1. First 2. Second 3. Third 4. >Fourth |  |
| **Section 2: General information about pregnancy and antenatal care** | | | | |
| 119 | | When you became pregnant with your last birth, was that pregnancy wanted (planned)? | \| 1. Wanted by then \| \| \|  \|  \| \| --- \| --- \| --- \| --- \| --- \| \| 1. Wanted late 2. Unwanted no more child \|  \|  \|  \|  \| |  |
| 120 | | Who was the main source of information about ANC care during your pregnancy? | 1. Television 2. Radio 3. Friends/relatives/neighbor 4. Health professionals 5. Internet 6. Other specify…………. |  |
| 121 | | Do you have a best friend who uses maternal care? *(antenatal care, delivery care or postnatal care*)? | 1. Yes 2. No | **If the answer is no skip to Q123** |
| 122 | | Does your best friend encourage you to use any of the maternal health services? | 1. Yes 2. No |  |
| 123 | | How often did you and your husband/partner together discuss the issue of using antenatal care during your pregnancy of last child? | 1. Very often (twice monthly) 2. Often (once monthly) 3. Less often (twice during pregnancy) 4. Seldom (once during pregnancy) 5. None 6. I didn’t have husband or partner |  |
| 124 | | what is your husband’s /partners attitude towards antenatal care? | 1. Positive 2. Negative 3. Neutral 4. I didn’t have husband or partner |  |
| 125 | | Did your partner/husband ever attend the antenatal care visit with you? | 1. Yes 2. No 3. I didn’t have husband or partner |  |
| 126 | | Did your partner /husband encourage you to follow ANC appropriately? | 1. Yes 2. No 3. I didn’t have husband or partner |  |
| 127 | | Did your partner/ husband remind your appointment to attend ANC? | 1. Yes 2. No 3. I didn’t have husband or partner |  |
| **Section 3: Antenatal care practice and quality of care** | | | | |
| 128 | Who did you see for antenatal care for your last pregnancy?  (more than one answer is possible) | | 1. Doctor/health officer 2. Nurse/midwife 3. Health extension worker 4. Don’t know |  |
| 129 | Did you attend antenatal care during any of your previous pregnancies before the pregnancy of most recent birth? (Cross check) | | 1. Yes 2. No 3. Never been pregnant before |  |
| 130 | Where did you receive antenatal care for the pregnancy of last birth? | | 1. Public Hospital 2. Health center 3. Health post 4. Private hospital/clinic 5. Non-governmental hospital/clinic |  |
| 131 | How do you rate the maximum waiting time at the health institution when you received care for the pregnancy of last birth? | | 1. Long 2. Moderate 3. Short 4. Do not remember |  |
| 132 | On pregnancy of last birth did you ever pay for antenatal care? | | 1. Yes 2. No | **If No, skip to Q134** |
| 133 | How do you rate the payment for antenatal care? | | 1. Expensive 2. Appropriate 3. Cheap 4. Don’t know |  |
| **Provider patient approach questions** | | | | |
| 134 | Maternal health care providers were respectful during provision of care | | 1. Agree 2. Neutral 3. Disagree |  |
| 135 | Maternal health care providers extend clear information regarding ANC. | | 1. Agree 2. Neutral 3. Disagree |  |
| 136 | I had a confidence on the skill of health care providers | | 1. Agree 2. Neutral 3. Disagree |  |
| 137 | The health care providers enable mothers to express freely their point of view regarding the care modalities. | | 1. Agree 2. Neutral 3. Disagree |  |
| 138 | I felt that my privacy and confidentiality was assured during my visits? | | 1. Agree 2. Neutral 3. Disagree |  |
| 139 | The health care providers show interest on what the mothers wants from the care. | | 1. Agree 2. Neutral 3. Disagree |  |
| 140 | The health care providers offer an opportunity to discuss and share making regarding the care. | | 1. Agree 2. Neutral 3. Disagree |  |
| 141 | I felt secured and confident when ever the health care provider touched me or being nearby. | | 1. Agree 2. Neutral 3. Disagree |  |
| 142 | The care providers recognize and respond to the emotion and feeling of the mother. | | 1. Agree 2. Neutral 3. Disagree |  |
| Maternal knowledge about Antenatal Care | | | | |
| 143 | What do you think about the importance of ANC? | | 1. Very important 2. Somewhat important 3. Not important 4. Do not know |  |
| 144 | When do you think a healthy pregnant woman should first start to attend antenatal care? | | 1. 1-3 months 2. 4-6 months 3. 7-9 months 4. Don’t know |  |
| 145 | How many times a healthy pregnant woman should visit ANC service? | | 1. Once 2. Two times 3. Three times 4. four and above |  |
| 146 | Are you aware of any dangerous pregnancy related symptoms? | | 1. Yes 2. No | **If “No” skip to Q148** |
| 147 | Can you mention some? (More than one answer is possible)  (Don’t read the options)  (circle all that applies) | | 1. Vaginal bleeding 2. Heavy vaginal fluid 3. Severe headache 4. Blurred vision 5. Fever 6. Abdominal pain 7. Persistent vomiting 8. face/hand swelling 9. Convulsions 10. Other (specify) |  |
| 148 | Are you aware of any dangerous pregnancy related complications? | | 1. Yes 2. No | **If “No” skip to Q 150** |
| 149 | Can you mention some? (More than one answer is possible)  (Don’t read the options)  (circle all that applies) | | 1. Hemorrhage 2. Hypertension 3. Obstructed labor 4. Abortion 5. Infection 6. Diabetes Mellitus 7. Obesity 8. Obstetric fistula 9. Anemia 10. Other (specify) |  |
| ANC service utilization | | | | |
| 150 | How many months pregnant were you at your **first** antenatal checkup during your pregnancy of most recent birth? | | 1. 1-3 months 2. ≥ 4 months |  |
| 151 | How many times in total did you receive antenatal care during your pregnancy of most recent birth? | | 1. Once 2. Twice 3. Three times 4. Four and more |  |
| 152 | Was your blood pressure measured at least once during ANC visit of last birth? | | 1. Yes 2. No 3. I don’t know |  |
| 153 | Were you weighed at least once during ANC visit of last birth?? | | 1. Yes 2. No 3. I don’t know |  |
| 154 | Was your height measured at least once during ANC visit of last birth? | | 1. Yes 2. No 3. I don’t know |  |
| 155 | Have you received iron supplement at least once during ANC visit of pregnancy of last birth? | | 1. Yes 2. No 3. I don’t know |  |
| 156 | Was blood test (blood type) done at least once during ANC visit of last birth? | | 1. Yes 2. No 3. I don’t know |  |
| 157 | Was urine test done at least once during ANC visit of last birth? | | 1. Yes 2. No 3. I don’t know |  |
| 158 | Was VDRL/syphilis test done at least once during ANC visit of last birth? | | 1. Yes 2. No 3. I don’t know |  |
| 159 | Was symphonies-fundus measurement done at least once during  ANC visit of pregnancy of last birth? | | 1. Yes 2. No 3. I don’t know |  |
| 160 | During your antenatal care visits for your pregnancy of most recent birth, did you ever have an injection in the arm to prevent against tetanus? | | 1. Yes 2. No 3. I don’t know |  |
| 161 | Have you tested for anaemia during ANC visit of pregnancy of last birth? | | 1. Yes 2. No 3. I don’t know |  |
| 162 | Was the Fetal heart rate measured at least once during ANC visit of pregnancy of last birth? | | 1. Yes 2. No 3. I don’t know |  |
| 163 | Was health providers’ counsel you about pregnancy related complications/danger signs during pregnancy at least once during ANC visit of last birth? | | 1. Yes 2. No 3. I don’t know |  |

**Section 4: House-hold assets**

| 164 | Does your household have? (circle if the item is available) | 1. Television 2. Radio 3. Electricity 4. Telephone 5. Refrigerator 6. Internet 7. Personal computer (laptop, desktop) |  |
| --- | --- | --- | --- |
| 165 | Does any of your household number own? | 1. Bajaj 2. Animal drown cart 3. Car 4. Motor bike |  |
| 166 | Who owns your household in which your household is living? | 1. Own 2. Rental 3. Other specify………. |  |
| 167 | Do you have separate rooms for sleeping? | 1. Yes 2. No |  |
| 168 | What is the main material of the wall in this dwelling? | \| 1. Natural (earth/sand) \| \| \| \|  \|  \|  \|  \| \| --- \| --- \| --- \| --- \| --- \| --- \| --- \| --- \| \| 1. Rudimentary (wood/bamboo) \| \| \| \| \| \| \| \| \| 1. Finished wall \|  \|  \|  \|  \|  \|  \|  \| \| 1. Other (specify) \| \| \| \| \| \| \| \| |  |
| 169 | What is the main material of the roof in this dwelling? | \| 1. Thatch (leaf) 2. Finished (concrete) 3. Corrugated iron 4. Other (specify) _______________ \| \|  \|  \|  \|  \|  \| \| --- \| --- \| --- \| --- \| --- \| --- \| --- \| \|  \|  \| \| \| \| \| \| |  |
| 170 | What is the main material of the floor in this dwelling? | \| 1. Natural (earth/sand) \|  \|  \|  \|  \| \| --- \| --- \| --- \| --- \| --- \| \| 1. Dung 2. Finished floor (cement, tile, brink) 3. Other (specify) \| \| \| \| \| \| |  |
| 171 | What is the main source of drinking water? | \| 1. piped water \| \| \| \| \| \| --- \| --- \| --- \| --- \| --- \| \| 1. open well 2. covered well / borehole \| \| \| \| \| \| 1. spring 2. Surface water (river, stream) 3. Rain water 4. Other (specify) \|  \|  \|  \|  \| \|  \|  \|  \|  \|  \| |  |
| 172 | What kind of toilet facility does members of your household use? | 1. No latrine/bush/ field 2. Traditional pit toilet 3. Ventilated pit latrine 4. Flush toilet 5. Other (specify) |  |
| 173 | Do you have a bed? | 1. Yes 2. No |  |
| 174 | Which source of energy you used for cooking? | 1. Electricity/stove 2. Wood/grass 3. Charcoal |  |
| 175 | Where do you prepare food? | 1. No building /outside 2. No separate building/at home 3. Have separate building/kitchen |  |
