# Supplementary material for: Magnitude and determinants of adequate antenatal care service utilization among mothers in Southern Ethiopia
Source: PLoS One. 2021 Jul 6;16(7):e0251477. doi: 10.1371/journal.pone.0251477 (PMC8259961; doi:10.1371/journal.pone.0251477)
Supplement: S2 File — (DOCX) [file pone.0251477.s002.docx]

| የክፍለ ከተማው ስም |  |
| --- | --- |
| የቀበሌው ስም |  |
| የቤተሰብ መለያ ቁጥር |  |

**ክፍል 1፤የማህበራዊ፣ ስነ ህዝባዊ እና ስነ ተዋልዶ ባህርያት**

| ተ.ቁ | መጠይቅ | መልሶች |  | |
| --- | --- | --- | --- | --- |
| 101 | እድሜሽ ስንት ነው? |  |  | |
| 102 | ከፍተኛ ያጠናቀቅሽው የት/ት ደረጃ ምንድነው? | 1. ያልተማረች 2. የመጀመርያ ደረጃ ት/ት (1-8) 3. የሁለተኛ ደረጃ ት/ት (9-12) 4. ሶስተኛ ደረጃ እና ከዛ በላይ(12^+^) |  | |
| 103 | ዋና ስራሽ ምንድነው? | 1. ስራ የሌላት 2. የመንግስት ሰራተኛ 3. የኢ-መንግስታዊተቃም ሰራተኛ 4. የግል ድርጅት ቅጥረኛ 5. የራስ ስራ 6. ሌላ ካለ ይግለፁ… |  | |
| 104 | ሃይማኖትሽ ምንድነው? | 1. ኦርቶዶክስ 2. ፕሮቲስታንት 3. አድቬንቲስት 4. ካቶሊክ 5. ሙስሊም 6. ሌላ ካለ ይግለፁ |  | |
| 105 | በአሁኑ ሰአት የትዳር ሁኔታሽ ምን ይመስላል? | 1. ያገባች 2. እጮኛ 3. ያላገባች 4. የሞተባት 5. የተፋታች 6. የተለያየች | መልሱ 'ያገባች' ፣እጮኛ ካልሆነ ወደ ጥያቄ ቁ.108 ይሂዱ | |
| 106. | የባለቤትሽ/የጉዋደኛሽ ዋና ስራው ምንድነው ? | 1. ስራ የሌለው 2. የመንግስት ሰራተኛ 3. የኢ-መንግስታዊተቃም ሰራተኛ 4. የግል ድርጅት ቅጥረኛ 5. የራስ ስራ 6. ሌላ ካለ ይግለፁ… |  | |
| 107 | ባለቤትሽ/ጉዋደኛሽ ያጠናቀቀው ከፍትኛ የት/ት ደረጃ ምንድነው? | 1. ያልተማረ 2. የመጀመርያ ደረጃ ት/ት (1-8) 3. የሁለተኛ ደረጃ ት/ት (9-12) 4. ሶስተኛ ደረጃ እና ከዛ በላይ (12^+^) |  | |
| 108 | በቤተሰባቹ ውስጥ የጤና ባለሞያ አለ? | 1. አለ 2. የለም |  | |
| 109 | ቴሌቪዝን/ራድዮ/ኢንተርኔት ለምን ያህል ግዜ ታዳምጫለሽ/ትመለከቻለሽ? | 1. በየቀኑ 2. በሳምንት አንዴ 3. በሳምንት ከአንድ ግዜ በታች 4. አዳምጨ/ተመልክቼ አላውቅም |  | |
| 110. | በቤተሰባቹ የጤና አገልግሎት በሚያስፈልግበት ግዜ የመጨረሻ ውሳኔ የሚሰጠው ማን ነው | 1. እኔ 2. ባለቤቴ 3. ሁለታችን በጋራ 4. ወላጆች 5. ሌላ ሰው…… |  | |
| 111 | በቤተሰባቹ አስተዳዳሪ/አባውራ ማን ነው? | 1. እኔ 2. ባለቤቴ 3. ሁለታችን በጋራ 4. ሌላ ሰው |  | |
| 112 | በመጨረሻ የእርግዝና ግዜ እድሜሽ ስንት ነበር? |  |  | |
| 113 | በጠቅላላ ስንት ግዜ አርገዘሻል? |  |  | |
| 114 | በጠቅላላ ስንት ግዜ ወልደሻል? |  |  | |
| 115 | በአሁኑ ሰአት በህይወት ያሉ ስንት ልጆች አሉሽ? |  |  | |
| 116 | ውርጃ አጋጥሞሽ ያውቃል? |  |  | |
| 117 | የሰባተኛ ወር እና ከዛ በላይ እርጉዝ ሁነሽ ወይም ልክ እንደተወለደ የሞተብሽ ልጅ አለ? |  |  | |
| 118 | መጨረሻ የወለድሽው ልጅ ላንቺ ስንተኛ ልጅሽ ነው? | 1. ኣንደኛ 2. ሁለተኛ 3. ሶስተኛ 4. ከ ኣራት በላይ |  | |
| 129 | የመጨረሻ ልጅሽን ስታረግዢ ፈልገሽው ነበር ? | 1. አዎ 2. ቆየት ብየ ማርገዝ ፈልጌ ነበር 3. በጭራሽ አልፈለኩትም ነበር |  | |
| 120 | ስለ የቅድመ ወሊድ ክትትል በዋነኝነት መረጃ ያገኘሽው ከየት ነው | 1. ከቴሌቪዝን 2. ከራድዮ 3. ከጎረቤት/Ùደኛ/ዘመድ 4. ከጤና ባለሞያ 5. ከጋዜጣ/መፅሄት 6. ሌላ ካለ ይግለፁ |  | |
| 121 | የእናቶች ጤና ክትትል(ቅድመ ወሊድ/ወሊድ/ድህረ-ወሊድ) በጤና ተÌም የምታደርግ በጣም የምትቀርቢያት ጉዋደኛ አለችሽ? | 1. አዎ 2. የለኝም | መልሱ 'አይ' ከሆነ ወደ ጥያቄ ቁ.123 ይሂዱ | |
| 122 | ጉዋደኛሽ የእናቶች ጤና ክትትል(ቅድመ ወሊድ/ወሊድ/ድህረ ወሊድ) እንድታደርጊ ታበረታታሻለች? | 1. አዎ 2. አይ |  | |
| 123 | ባለቤትሽ/ፍቅረኛሽ ጋራ የመጨረሻ ልጅሽን ስታረግ¢ ስለ የቅድመ ወሊድ ክትትል ጉዳይ ለምን ያህል ግዜ ትወያዩ ነበር? | 1. ሁለት ገዜ በወር 2. በየወሩ 3. እርጉዝ በነበርኩበት ሁለት ግዜ ብቻ 4. እርጉዝ በነበርኩበት አንድ ግዜ ብቻ 5. ምንም ተወያይተን አናውቅም 6. ባለቤት/ፍቅረኛ የለኝም |  | |
| 124 | ባለቤትሽ/ፍቅረኛሽ ስለ የቅድመ ወሊድ ክትትል ያለው አመለካከት ምን ይመስላል? | 1. ጥሩ 2. ጥሩ አይደለም 3. መካከለኛ 4. ባለቤት/ፍቅረኛ የለኝም 5. አለውቅም |  | |
| 125 | ባለቤትሽ/ፍቅረኛሽ የቅድመ ወሊድ ክትትል ስታደርጊ አብሮሽ ሄዶ ያውቃል? | 1. አዎ 2. አይ 3. ባለቤት/ፍቅረኛ የለኝም |  | |
| 126 | ባለቤትሽ/ፍቅረኛሽ የቅድመ ወሊድ ክትትል በትክክል እንድታደርጊ ያበረታታሽ ነበር? | 1. አዎ 2. አይ 3. ባለቤት/ፍቅረኛ የለኝም |  | |
| 127 | ባለቤትሽ/ፍቅረኛሽ የቅድመ ወሊድ ክትትል ቀጠሮ ቀንሽን እንዳትረሺ ያግዝሽ ነበር? | 1. አዎ 2. አይ 3. ባለቤት/ፍቅረኛ የለኝም |  | |
| 128 | የመጨረሻ ልጅሽን ስታረግዢ የቅድመ ወሊድ ክትትል ያደረገልሽ ማን ነበር? | 1. ሀኪም/ጠና መኮንን/ 2. ነርስ/አዋላጅ ነርስ 3. የጤና እክስቴንሽን ሰራተኛ 4. አላውቅም |  | |
| 129 | ከዚህ በፊት በነበረው የእርግዝናሽ ግዜ የቅድመ ወሊድ ክትትል አድርገሽ ነበር? | 1. አዎ 2. አላደረግኩም 3. ከዚህ በፊት አላረገዝኩም |  | |
| 130 | የመጨረሻ ልጅሽን እርጉዝ በነበርሽበት ወቅት የቅድመ ወሊድ ክትትል ያደረግሽው የት ነው? | 1. የመንግስት ሆስፒታል 2. ጤና ጣብያ ሆስፒታል 3. ጤና ኬላ ሆስፒታል 4. የግል ሆስፒታል 5. መንግስታዊ ያልሆነ |  | |
| 131 | ወደ ጤና ተቁዋም ከደረስሽ በሁላ የምትፈለጊውን አገልግሎት ለማግኘት የምትጠብቂው ግዜ እንዴት ታዪዋለሽ ? | 1. ረዘም ያለ ነበር 2. መካከለኛ ነበር 3. አጭር ነበር 4. ኣላስታውስም |  | |
| 132 | የመጨረሻ ልጅሽን እርጉዝ በነበርሽበት ወቅት ለቅደመ ወሊድ ክትትል ከፍለሽ ታውቂያለሽ? | 1. አዎ 2. አይ | መልሱ 'አይ' ከሆነ ወደ ጥያቄ ቁ.134 ይሂዱ |  |
| 133 | በንቺ አስተሳሰብ ለቅደመ ወሊድ ክትትል የሚከፈለው ክፍያ እንዴት ታዪዋለሽ? | 1. ውድ ነበር 2. በኣግባቡ ነበር 3. ርካሽ ነበር |  | |
| 134 | በመጨረሻው ልጄ እርግዝና የቅድመ ወሊድ ክትትል ወቅት የጤና ባለሙያወቹ የነበራቸው መስተንግዶ በኣግባቡ እና በ ኣክብሮት ነበር | 1. እስማማለው 2. ኣስተያየት የለኝም 3. ኣልስማማም |  | |
| 135 | በመጨረሻው ልጄ እርግዝና የቅድመ ወሊድ ክትትል ወቅት የጤና ባለሙያወቹ ለደንበኞቻቸው መረጃ የሚሰጡበት መንገድ ግልጽ ና ቀላል ነበር። | 1. እስማማለው 2. ኣስተያየት የለኝም 3. ኣልስማማም |  | |
| 136 | በመጨረሻው ልጄ እርግዝና የቅድመ ወሊድ ክትትል ወቅት የጤና ባለሙያውቹ ክህሎት ኣላቸው የሚል እምነት ኣለኝ | 1. እስማማለው 2. ኣስተያየት የለኝም 3. ኣልስማማም |  | |
| 137 | በመጨረሻው ልጄ እርግዝና የቅድመ ወሊድ ክትትል ወቅት፣የፈለኩትን ጥያቄ እና ኣስተያየት ለጤና ባለሞያው መጠየቅ እችል ነበር | 1. እስማማለው 2. ኣስተያየት የለኝም 3. ኣልስማማም |  | |
| 138 | በመጨረሻው ልጄ እርግዝና የቅድመ ወሊድ ክትትል ወቅት የጤና ባለሙያው ሚስጥሬን ጠብቆልኝል ብዬ ኣስባለው። | 1. እስማማለው 2. ኣስተያየት የለኝም 3. ኣልስማማም |  | |
| 139 | በመጨረሻው ልጄ እርግዝና የቅድመ ወሊድ ክትትል ወቅት የጤና ባለሙያው ፍላጎቴን ለማዳመጥ ዝግጁ ነበር | 1. እስማማለው 2. ኣስተያየት የለኝም 3. ኣልስማማም |  | |
| 140 | በመጨረሻው ልጄ እርግዝና የቅድመ ወሊድ ክትትል ወቅት የጤና ባለሙያው በኣገልግሎቱ ዙርያ ውሳኔወችን እንድወስን እና ኣስተያየት እንድሰጥ እድሉን ይሰጠኝል | 1. እስማማለው 2. ኣስተያየት የለኝም 3. ኣልስማማም |  | |
| 141 | በመጨረሻው ልጄ እርግዝና የቅድመ ወሊድ ክትትል ወቅት የጤና ባለሙያው ሲነካኝም ሆነ ሲጠጋኝ እተማመንበታለው ኣልረበሽም | 1. እስማማለው 2. ኣስተያየት የለኝም 3. ኣልስማማም |  | |
| 142 | በመጨረሻው ልጄ እርግዝና የቅድመ ወሊድ ክትትል ወቅት የጤና ባለሙያው ስሜቴን እና ሃሳቤን ይረዳልኝል ኣልፎም በኣግባቡ ምላሽ ይሰጠኛል | 1. እስማማለው 2. ኣስተያየት የለኝም 3. ኣልስማማም |  | |
| 143 | የቅድመ ወሊድ ክትትል ጠቃሚነቱን እነዴት ትገለጭዋለሽ? | 1. እስማማለው 2. ኣስተያየት የለኝም 3. ኣልስማማም |  | |
| 144 | አንድ እናት የመጀመርያ የቅድመ ወሊድ ክትትል መጀመር ያለባት በስንተኛው ወር ነው | 1. 1-3 ወር 2. 4-6 ወር 3. 7-9 ወር 4. አላውቅም |  | |
| 145 | አንድ እናት እስክትወልድ ድረስ ስንት ግዜ የቅድመ ወሊድ ክትትል ማድረግ ይኖርባታል | 1. አንድ ግዜ 2. ሁለት ግዜ 3. ሦስት ገዜ 4. አራት እና ከዛ በላይ |  | |
| 146 | በእርግዝና ግዜ ሊከሰቱ የሚችሉ የተለመዱ ምልክቶች ምን ምን እንደሆኑ ታውቂያለሽ | 1. አዎ 2. አይ | መልሱ 'አይ' ከሆነ ወደ ጥያቄ ቁ.148 ይሂዱ | |
| 147 | በእርግዝና ግዜ ሊከሰቱ የሚችሉ የተለመዱ ምልክቶች ምን ምን ናቸው | 1. ደም መፍሰስ 2. የማህፀን ፈሳሽ 3. ከባድ የራስ ምታት 4. የእይታ መደበዝዘ 5. ትኩሳት 6. የሆድ ቁርጠት 7. የማያቃርጥ ትውከት 8. የእጅ እና የፊት ማበጥ 9. መንቀጥቀጥ 10. ሌላ ካለ ይገለፅ……. |  | |
| 148 | ከእርግዝና ጋር ተያይዘው የሚከሰቱ በሽታዎች ምን ምን እንደሆኑ ታውቂያለሽ | 1. አዎ 2. አይ | መልሱ 'አይ' ከሆነ ወደ ጥያቄ ቁ.150 ይሂዱ | |
| 149 | ከእርግዝና ጋር ተያይዘው የሚከሰቱ በሽታዎች ምን ምን ናቸው | 1. ከወሊድ በሃላ ደም መፍሰስ 2. የደም ግፊት. 3. የምጥ ሰአት መርዘም 4. ውርጃ 5. ኢንፌክሽን 6. የስካር በሽታ 7. ከመጠን በላይ የሆነ ውፍረት 8. ፊስቱላ 9. የደም ማነስ. 10. ሌላ ካለ ይግለፁ…… |  | |
| 150 | በመጨረሻው ልጀሽ የእርግዝናሽ ወቅት የቅድመ ወሊድ ክትትል የጀመርሽው በስንተኛው ወር ነው | 1. 1-3 ወር 2. ≥ 4 ወር |  | |
| 151 | በመጨረሻው ልጀሽ የእርግዝና ወቅት ስንት ግዜ የቅድመ ወሊድ ክትትል አድርገሻል | 1. አንድ ግዜ 2. ሁለት ግዜ 3. ሦስት ግዜ 4. አራት ግዜ እና ከዛ በላይ |  | |
| 152 | በመጨረሻው ልጀሽ እርግዝና የቅድመ ወሊድ ክትትል በምታደርጊበት ወቅት የደም ግፊትሽ ተለክቶ ነበር | 1. አዎ 2. አይ 3. ኣላውቅም |  | |
| 153 | በምጨረሻው ልጀሽ እርግዝና የቅድመ ወሊድ ክትትል በምታደርጊበት ወቅት ክብደት ተለክተሸ ነበር | 1. አዎ 2. አይ 3. ኣላውቅም |  | |
| 154 | በጨረሻው ልጀሽ እርግዝና የቅድመ ወሊድ ክትትል በምታደርጊበት ግዜ ወቅት ቁመትሽ ተለክተሸ ነበር | 1. አዎ 2. አይ 3. ኣላውቅም |  | |
| 155 | በጨረሻው ልጀሽ እርግዝና የቅድመ ወሊድ ክትትል በምታደርጊበት ወቅት የደም ማነስ መድሃኒት/ አይረን እንክብል ተሰጥቶሻል/ገዝተሸል | 1. አዎ 2. አይ 3. ኣላውቅም |  | |
| 156 | በጨረሻው ልጀሽ እርግዝና የቅድመ ወሊድ ክትትል በምታደርጊበት ወቅት የደም አይነት ምርመራ ተደርጎልሽ ነበር | 1. አዎ 2. አይ 3. ኣላውቅም |  | |
| 157 | በጨረሻው ልጀሽ እርግዝና የቅድመ ወሊድ ክትትል በምታደርጊበት ወቅት የሽንት ምርመራ ተደርጎልሽ ነበር | 1. አዎ 2. አይ 3. ኣላውቅም |  | |
| 158 | በጨረሻው ልጀሽ እርግዝና የቅድመ ወሊድ ክትትል በምታደርጊበት ወቅት የአባላዘር ምርመራ ተደርጎልሽ ነበር | 1. አዎ 2. አይ 3. ኣላውቅም |  | |
| 159 | በጨረሻው ልጀሽ እርግዝና የቅድመ ወሊድ ክትትል በምታደርጊበት ወቅት የእርግዝናው መጠን በሜትር/በእጅ ተለክቶልሽ  ነበር | 1. አዎ 2. አይ 3. ኣላውቅም |  | |
| 160 | በጨረሻው ልጀሽ እርግዝና የቅድመ ወሊድ ክትትል በምታደርጊበት ወቅት በክንድ ላይ በመርፌ የሚሰጥ የመንጋጋ ቆልፍ ክትባት ወስደሻል | 1. አዎ 2. አይ 3. ኣላውቅም |  | |
| 161 | በጨረሻው ልጀሽ እርግዝና የቅድመ ወሊድ ክትትል በምታደርጊበት ወቅት የደም ማነስ ምርመራ ተደርጎልሽ ነበር | 1. አዎ 2. አይ 3. ኣላውቅም |  | |
| 162 | በጨረሻው ልጀሽ እርግዝና የቅድመ ወሊድ ክትትል በምታደርጊበት ወቅት የልጅሽ የልብ ምት ተለክቶ ነበር | 1. አዎ 2. አይ 3. ኣላውቅም |  | |
| 163 | በጨረሻው ልጀሽ እርግዝና የቅድመ ወሊድ ክትትል በምታደርጊበት ወቅት ከእርግዝና ጋር ተያይዘው ሊመጡ ስለሚችሉ የጠና እክሎች/በሽታዎች መረጃ ተሰጥቶሽ ነበር | 1. አዎ 2. አይ 3. ኣላውቅም |  | |

**ክፍል 4፡ የቤተስብ የሀብት ሁኔታ በተመለከተ**

|  | **የቤተሰብ መረጃ** | **ምላሽ** |  |
| --- | --- | --- | --- |
| 164 | በቤታቹ ውስጥ ከሚከተሉት ውስጥ የትኞቹ ናቸው ያሉት? | 1. ቴሌቪዥን 2. መብራት 3. ራድዮ 4. ስልክ 5. የግል ኮምፒውተር 6. ኢንተርኔት 7. ፍሪጅ 8. ጀነሬተር |  |
| 165 | ቤተሰባቸሁ ከሚከተሉት ውስጥ የትኛው አለው | 1. ባጃጅ 2. ጋሪ 3. መኪና 4. ሞተር |  |
| 166 | የምትኖሩበት መኖርያ ቤት የማን ነው? | 1. የኪራይ 2. የራሳችን 3. ሌላ |  |
| 167 | የተለየ መኝታ ክፍል ኣላችሁ ወይ? | 1. ኣዎ 2. የለንም |  |
| 168 | የቤታቹ ግድግዳው ከምንድነው የተሰራው? | 1. ተፈጥሮኣዊ (አፈር ፣አካባቢያዊ ጡብ) 2. እንጨት፣ ቀርከሃ 3. ሲሚንቶ 4. ሌላ / ማብራሪያ … |  |
| 169 | የቤታቹ ውስጠኛው ኮርኒስ ከምንድነው የተሰራው | 1. ከሳር 2. ቆርቆሮ 3. ከ ሲሚንቶ 4. ሌላ ካለ ግለጭ……. |  |
| 170 | የቤት ወለል ከምንድነው የተሰራው ? | 1. ተፈጥሮኣዊ ( አፈር/ አሸዋ/ቀርከሃ ) 2. እበት 3. ያለቀለት (የሴራሚክ ንጣፎች ፣ሲሚንቶ 4. ሌላ …………………… |  |
| 171 | አዘውትረችሁ የምትጠቀሙት የመጠጥ ውሃ ምንጭ የቱ ነው? | 1. ቧንቧ ውሃ 2. ክፍት የ ጉድጓድ ውሃ 3. የተከደነ ጉድጓድ ውሃ 4. የምንጭ ውሃ 5. ወራጅ ውሃ 6. የዝናብ ውሃ 7. ሌላ………………… |  |
| 172 | ምን ዓይነት የመፀዳጃ ቤት አለችሁ? | 1. የለንም/ሜደ ላይ 2. ስላብ ያለው መፀዳጃ ቤት, 3. በሲሚንቶ የተሰራ መፀዳጃ ቤት 4. ዘመናዊ መፀዳጃ ቤት 5. ሌላ ካለ ግለጪ ____________ |  |
| 173 | የቤት አልጋ አላችሁ? | 1. አዎ 2. የለንም |  |
| 174 | አዘውትራችሁ የምትጠቀሙት የማብሰያ ዘዴዎች ? | 1. የኤሌክትሪክ ኃይል 2. ቁጥቋጦ /ሳር/እንጨቶች 3. ከሰል 4. ሌላ ___ |  |
| 175 | ምግብ የምታበስሉት የት ነው? | 1. በቤት ውስጥ አይሰራም/ዉጭ ላይ ነው 2. በተለየ ሕንፃ/ኩሽና 3. በቤት ውስጥ |  |
