# Supplementary material for: Magnitude and determinants of adequate antenatal care service utilization among mothers in Southern Ethiopia
Source: PLoS One. 2021 Jul 6;16(7):e0251477. doi: 10.1371/journal.pone.0251477 (PMC8259961; doi:10.1371/journal.pone.0251477)
Supplement: Attachment — Submitted filename: response to reviewers.docx [file pone.0251477.s005.docx]

**Manuscript Title:** Magnitude and determinants of antenatal care service utilization among mothers in Southern Ethiopia 2019.

**Dear editor and reviewers**

We would like to extend our heartfelt gratitude and appreciation for your valuable comments and priceless time. Thank you, your comments have helped us a lot to improve the manuscript. We tried to address all comments point by point in this paper.

# Responses for the editor

1. Please ensure that your manuscript meets PLOS ONE's style requirements, including those for file naming.

Response: Comment accepted and the manuscript has been revised including figures and tables to meet PLOS ONE's style requirements

2. Please include additional information regarding the questionnaire used in the study and ensure that you have provided sufficient details that others could replicate the analyses. For instance, if you developed a questionnaire as part of this study and it is not under a copyright more restrictive than CC-BY, please include a copy, in both the original language and English, as Supporting Information.

Response: Comment accepted and changes have been made, please see the highlighted manuscript from line (159-170). And the tool we used for this study was submitted as supplementary material both in English and Amharic language.

3. In the Methods, please describe how the questionnaire was validated. If this did not occur, please provide the rationale for not validating the questionnaire

Response: The questionnaire was validated in the following way. After the initial pool of questionnaire items were written, qualified experts reviewed the items to make sure they are accurate, free of item construction problems, and grammatically correct. Before the study was conducted in the intended participants pretest was conducted on 5% of sample size to clear confusion about any items, and whether respondents have suggestions for possible improvements of the items. We analysed responses to the pilot and feedback from our interviewers on interviewees’ level of understanding and any questions that caused interviewee confusion or difficulty. We then used this information to revise the questionnaire so as to further ensure clarity and comprehensibility.

4. Please remove any funding-related text from the manuscript and let us know how you would like to update your Funding Statement.

Response: Comment accepted and amendment has been done. We need the funding statement to be read as “Wolaita Sodo University covers perdium for data collectors and supervisors” and this funding statement was included in the cover letter.

5. We suggest you thoroughly copyedit your manuscript for language usage, spelling, and grammar. If you do not know anyone who can help you do this, you may wish to consider employing a professional scientific editing service.

Response: Comment accepted and amendment has been done. We tried to revise the whole document.

# Response to reviewer 1

1. I advise to change the topics to Magnitude and determinants of adequate antenatal care utilization among mothers in Southern Ethiopia.

Response: Comment accepted and changes have been made. Please see the highlighted manuscript from line (1-3)

2. Better to report factor associated with adequate antenatal care utilization with OR with 95% CI

Response: Comment accepted and amendment done (please see the highlighted manuscript line (32-39).

3. The introduction to the study appears good. Authors should briefly explain what has been done so far. To be more interesting, author need to consider “what is the additional knowledge has this study is going to generate”? Try to explain the gap of the topic and the reason why you performed this research

Response: Comment accepted and changes made. The introduction part was modified to show the gap and to make strong justification. (please see the highlighted manuscript line (80-110).

4. How to validate your tool?

Response: The questionnaire was validated in the following way. After the initial pool of questionnaire items were written, qualified experts reviewed the items to make sure they are accurate, free of item construction problems, and grammatically correct. Before the study was conducted in the intended participants pretest was conducted on 5% of sample size to clear confusion about any items, and whether respondents have suggestions for possible improvements of the items. We analysed responses to the pilot and feedback from our interviewers on interviewees’ level of understanding and any questions that caused interviewee confusion or difficulty. We then used this information to revise the questionnaire so as to further ensure clarity and comprehensibility.

5. In the result section you present data related to knowledge and perception, how to measure knowledge and perception?

Response: knowledge and perception measurement used for the study were included in table 1. (please see the highlighted manuscript page (31-32)

6. What are unique variables/factors/ examined compared to the pervious available studies. All identified variables are already addressed previously studies. Therefore, include variable should not be redundant

Response: Analyzing all the three vital ANC adequacy indicators with in a single study in line with WHO recommendation of adequate ANC makes this study unusual. Besides, to our best knowledge, even though there are some researchs conducted in Ethiopia similar with this study; all significant variables were not incorporated and analyzed together in a single study. The result from those studies may miss some potential independent variables and may not deliver a detailed picture on illustrating a comprehensive conceptual framework. Whereas in this study almost all important variables were systematically comprised which were bypassed in other researches.

Furthermore, despite service content might be influenced by the service providers’ approach it has not been addressed previously.

7. The authors do not explain how they checked for multicollinearity. if the author plan to take p-value 0.2, as a cut of point for multivariable analysis, the author must present the result of identified variable with their p-value at bivariate model

Response: Multicollinearity was checked by variable inflation factor and all showed no multicollinearity with VIF of less than five. Depending on the comments received from reviewers the outcome variable was dichotomized and logistic regression model was applied. We take p-value of 0.05 as cut off point for bivariate model. Those significant variables identified in bivariate model were presented in Table 4. (please see the highlighted manuscript page 36-37)

8. A result needs to improve the flow of sections. Tables are not standard, there is boarding information

Response: The overall flow of result section and the table quality is improved according to the journal guidelines (please see the overall result section and table in the highlighted manuscript).

9. The discussion part is good, but you have to formulate the clinical and public health implications of the study findings. What are the innovative ideas, for scale up and ensure quality and safe services?

Response: Comment accepted and amendments done. We include the clinical and public health implications of the finding. (Please see the highlighted manuscript page (22-23), and line (449-474).

Analyzing all the three vital ANC adequacy indicators with in a single study in line with WHO recommendation of adequate ANC makes this study unusual. Besides, to our best knowledge, even though there are some researchers conducted in Ethiopia similar with this study; all significant variables were not incorporated and analyzed together in a single study. The result from those studies may not deliver a detailed picture on illustrating a comprehensive conceptual framework. Whereas in this study almost all important variables were systematically comprised which were bypassed in other researches.

Furthermore, despite service content might be influenced by the service providers’ approach it has not been addressed previously.

10. In study, only 23% of participants utilized all three ANC attributes adequately. This finding was not in line with the national standard, ----What was national figure? Is it possible to compare with the national data? You said---However, during this study, although the magnitude of adequate utilization was very low, --what is your assumption to say very low? It was moreover higher than the previous study done in Ethiopia only 2.6 % and 11% of participants received overall adequate ANC [24, 34].

Response: We totally amend the paragraph in the discussion section (please see in the highlighted manuscript line number (358-362).

11. Authors can suggest some recommendation, but not make a hard conclusion that those strategies would work or hinder.

Response: We totally amend the conclusion part (please see in the highlighted manuscript in line number (476-481).

12. The use of tablets with digital survey tools for the data collection and ordinal regression analysis was the strength of this study. Do you think; is it the strength of study? On the other hand, due to the cross-sectional nature of the study, it is difficult to establish a temporal relationship between the determinants and outcome variables. It is already known facts, better to acknowledge other source of bias as a limitation of study.

Response: We totally amend the strength and limitation part (please see in the highlighted manuscript in line number (438-448).

# Response to reviewer #2

1. My major concern is the method section, where the authors defined the dependent variable of “adequate antenatal care utilization” as three composite attributes. The third attribute of “service content” was based on information obtained from the study participants, when indeed; it was clearly evident that some of the identified antenatal care components cannot be measured adequately by merely asking the participants. It was also difficult to identify what the standard measure of adequate antenatal care utilization was after using the 3 measures and how this was used in the eventual data analysis. The data analysis section is weak and does not seem to synchronize with the objectives and methodologies described in the paper.

Response: Comment accepted and we already mentioned it as a limitation of the study. But the analysis section is totally modified. Please see the highlighted manuscript line (189-211) and (443-446)

2. The paper is very poorly written with poor English language and disorderly presentation. Perhaps, an English language editor should edit the paper before it can be suitable for publication.

Response: Comment accepted and we tried to revise the whole document.

3. The introduction needs to be stronger and make a case to justify the novelty and justification for the study.

Response: Comment accepted and the introduction section is totally modified. Please see the highlighted manuscript line (80-110).

# Response to reviewer #3

1. Last paragraph: The justification, and indeed the entire paragraph, needs to be re-written for clarity.

Response: Comment accepted and the entire section of introduction is modified. (Please see the highlighted manuscript from line (80-110).

2a. Source of data and data collection methods: Anderson Newman framework for healthcare utilization doesn’t have a questionnaire that can be adapted; the theory only provided some concepts that can be adapted. Hence, authors should rephrase the first statement under this sub heading.

Response: We rephrase the whole sentences. Please see the manuscript line number (159-161).

2b. Data quality control: The last paragraph appears cumbersome; what type of inconsistencies are the authors referring to in “line 6” of that paragraph, also what type of transformation did they do? How were the missing values mentioned in “line 7”managed? Is the questionnaire self or interviewer administered?

Response: After the data collector made the interview, the supervisor repeated the interview on 10% of sample size. The principal investigator then double checks both responses gained from interviews with data collectors and supervisor’s weather there is any difference (inconsistency) in the response of similar respondent.

*Before analysis we made transformation of many variables like age (from continues variable grouped into three category”15-24”,”25-34” and 35-49”, marital status(from married, cohabiting (living together), single, widowed and divorced dichotomized to “married” and “single”, different household assets to wealth index using Principal component factor analysis,…..

*The questionnaire was interviewer administered.

* Missing values was managed in different ways. The first measure we take to prevent the occurrence of missing value related with unfilled questions by the data collector was using tablets to collect data. As questions in the tablet were arranged to not allow passing of the question without required answer except those questions with skipping nature, this prevents missing value occurred by data collector (unintentional passing of the question without asking the question /writing the response). Additionally we used multiple imputation to manage missing value by random. Please check submitted data before and after multiple imputations.

2c. Authors need to provide further details regarding:

i. How the data was handled?

Response: Strategies used to handle this data was mentioned in the manuscript section of “source of data and data collection methods” and “Data quality control “. Please see the highlighted manuscript line (159-187).

ii. The instrument used and how validity and reliability were ensured

**Response**: The questionnaire was validated in the following way. After the initial pool of questionnaire items were written, qualified experts reviewed the items to make sure they are accurate, free of item construction problems, and grammatically correct. Before the study was conducted in the intended participants pretest was conducted on 5% of sample size to clear confusion about any items, and whether respondents have suggestions for possible improvements of the items. We analysed responses to the pilot and feedback from our interviewers on interviewees’ level of understanding and any questions that caused interviewee confusion or difficulty. We then used this information to revise the questionnaire so as to further ensure clarity and comprehensibility. Additionally, item questions were checked for reliability with cronbach’s alpha of 0.976.

2d.

i. The outcome variable should simply be termed ‘ANC utilization’. This will remove the confusion between ‘ANC utilization’ as a variable and ‘adequate ANC utilization’ as one of the categories of that variable. Also, the four categories, as presented in the results, should be clearly defined in the methods.

Response: Comment accepted and amendments done (please see the highlighted manuscript line (130-145).

ii. ‘Service contents’ was one of attributes used to construct the outcome variable. This presents a challenge with regards to the choice of explanatory or predictor variables (Table 1). The number of service contents received by a mother is essentially not within her domain of control. Such will depend on distal factors i.e. factors related to the health care providers and health care facilities. However, these variables were not included by the authors in their list of explanatory variables as contained in Table 1. This appears to be a major omission. It is recommended that, if the authors obtained any such data regarding health care provider / facility factors, such should be included as explanatory variables in the bivariate and multivariate analyses aimed at identifying predictors of ‘adequate ANC utilization’.

Response: Comment accepted and amendments done. Despite factors related with health care providers and health care facility variables were assessed, they were not included in table 1. We improved the contents in table 1….please see the highlighted manuscript page (31-32).

e. Data processing and analysis

i. As stated by authors, all the assumptions for ordinal logistic regression (OLR) were considered. Were they met? If yes, it should be stated. What were the chi square statistics and p-value for the test of proportional odds (or test of parallel lines) for the model?

Response: Depending on the comments received from reviewers the model is changed to logistic regression model.

ii. The use of OLR poses some problems within the context of authors’ intention and the eventual interpretation of the results. The objective was to identify predictors of ‘overall adequate ANC utilization’, which is a discrete category, the highest among the four (4) different categories of the outcome variable. The OLR may not be the most appropriate for this purpose, even though the outcome variable appears to be ordinal in ranking. Ideally, OLR does not model the effect of explanatory variables on a discrete category or outcome, but rather their effect on the probability of increase (or decrease) across the different thresholds (or categories) of the ordinally ranked outcome variable. Two options are hereby recommended:

*a. Authors should revise the presentation of the results to reflect the proper interpretation of an OLR model. The reference category of the outcome variable must also be stated. In that case, the authors will be reporting the odds of obtaining higher levels of the categories of ANC utilization.

*b. Alternatively, to predict ‘overall adequate ANC utilization’ as a discrete category, there may be the need to dichotomize the outcome variable and apply the binary logistic regression. If authors prefer to retain the four categories, the use of multinomial regression should be considered

Response: Comment accepted and the outcome variable is dichotomized and logistic regression model is applied

3. Results
i. Knowledge and perception: How were these assessed, rated and/or categorized? The methods section provided no information about these variables.

Response: Comment accepted and amendment done. Please see the highlighted manuscript Table 1, page (31-32).

ii. Levels of adequate utilization: The descriptive results of each of the 3 constituent variables i.e. frequency of visits, timing and service content, should be presented as a prelude to that of the composite ‘adequate ANC utilization’.

Response: comment accepted and the results were presented (please see the result section of the highlighted manuscript line (240-244) and figure 1.

4. Grammar editing: The article will benefit greatly from grammar editing and this will help the readability of the article

Response: Comment accepted and amendment has been done by using premium grammerly.

MINOR ISSUES

1. The literature was treated fairly however there is a document by WHO which may be of interest to the authors “Trends in maternal mortality 2000 to 2017: estimates by WHO, UNICEF, UNFPA, World Bank Group and the United Nations Population Division: executive summary” <https://apps.who.int/iris/handle/10665/327596>

Response: Thank you. We tried to see the document and some amendments done based on it.

2. Methods
i. The population projection for Ethiopia as mentioned in line 4 should be properly referenced.

Response: comment accepted and amendment made. Please see the highlighted manuscript line 117.

ii. Outcome variable –Service content: The authors should please clarify their statement “…over or up to mean (8) of 12…”

Response: Comment accepted and amendment done. The whole sentences were rewritten. Please see the highlighted manuscript line 144.

iii. Sampling procedure: The sentence in line 5, starting with Households were enrolled by systematic random sampling after identified eligible women…” is unclear, the authors should clarify to ensure that the readers understand what the authors did exactly.

Response: Comment accepted and the whole sentence is rewritten. Please see in highlighted manuscript in line (154-156).

3. In paragraph 1 under result section, authors reported religion but this was not shown on table 2 as indicated

Response: Comment accepted and amendment has been done. Please see table 2 in the highlighted manuscript page 33.

4. A few of the literature used to discuss the findings are not similar with the results they are being compared with especially in the aspect of definition of utilization

Response: Comment accepted. As there are not enough studies conducted similar with this study it was difficult to compare but some modification was done. Please see the discussion section of highlighted manuscript.

5. The authors repeated results and made recommendations in the conclusion section rather than presenting a specific conclusion.

Response: We totally amend the conclusion part. Please see in the highlighted manuscript line (476-481).

6. The authors should pay attention to the list of references and be consistent with the journal’s format for managing references.

Response: Comment accepted and we tried to list all references with journals format (Vancouver style)
