# Supplementary material for: Magnitude and determinants of adequate antenatal care service utilization among mothers in Southern Ethiopia
Source: PLoS One. 2021 Jul 6;16(7):e0251477. doi: 10.1371/journal.pone.0251477 (PMC8259961; doi:10.1371/journal.pone.0251477)
Supplement: Attachment — Submitted filename: Response to reviewers.docx [file pone.0251477.s006.docx]

**Manuscript Title:** Magnitude and determinants of antenatal care service utilization among mothers in Southern Ethiopia 2019.

**Dear editor and reviewers**

We would like to extend our heartfelt gratitude and appreciation for your valuable comments and priceless time. Thank you, your comments have helped us a lot to improve the manuscript. We tried to address all comments point by point in this paper.

**Responses for the editor**

1. In the revised version, you have incorporated most of the comments of reviewers/editor. Nevertheless, I will suggest including figures from the table in the text of findings section on line 252-268

Response: Comment accepted and the manuscript has been revised including figures from the table, please see the highlighted manuscript from line (271-316)

2. Please review your reference list to ensure that it is complete and correct. If you have cited papers that have been retracted, please include the rationale for doing so in the manuscript text, or remove these references and replace them with relevant current references. Any changes to the reference list should be mentioned in the rebuttal letter that accompanies your revised manuscript. If you need to cite a retracted article, indicate the article’s retracted status in the References list and also include a citation and full reference for the retraction notice.

**Response**: No retracted manuscript was cited. But some changes have been made to the reference, because there were mistakenly cited references. So, we made replacement to some references, please see the highlighted manuscript from line (577-604)

- We also remove two references because of redundancy, please see the highlighted manuscript line 651 and 680

**Response to reviewer #1**

**Abstract**

1. Result: better to narrate the finding line with the objective of the study

Response: comment accepted and amendment done, please see the highlighted manuscript from line (34-42)

2. Conclusion: better to recommend specific action

Response: comment accepted and amendment done, please see the highlighted manuscript from line (43-46)

3. Authors extensively edited the manuscript and there is great improvement. Authors responded the comments point by point, but some of the comments are not well addressed as it stated.

**Response:** Thank you for your comment; we tried to revise the whole revised manuscript line by line to address all the comments given

**Response to reviewer #3**

1. On table 5, the abbreviations that were listed under the key were not presented on the table. Please Check and correct appropriately

Response: comment accepted and amendment done. Please see the highlighted manuscript from line (747-749)

2. Check the spelling of category under the key, it is written as "catagory"

Response: Comment accepted and amendment done. Please see the highlighted manuscript line 747

3. Grammar editing: there is still more to be done, I don't think Grammarly alone is sufficient here, the authors should please get a native speaker to help proof read the manuscript.

Response: Comment accepted and amendment has been done. We tried to improve the readability of the manuscript by using native speaker.
